# Whole-Proteome Analysis of Twelve Species of Alphaproteobacteria Links Four Pathogens

**DOI:** 10.3390/pathogens2040627

**Published:** 2013-11-26

**Authors:** Yunyun Zhou, Douglas R. Call, Shira L. Broschat

**Affiliations:** 1 School of Electrical Engineering and Computer Science, Washington State University, Pullman, WA 99164, USA; E-Mails: zhouyunyun11@gmail.com (Y.Z.); drcall@vetmed.wsu.edu (D.R.C.); 2 Paul G. Allen School for Global Animal Health, Washington State University, Pullman, WA 99164, USA; 3 Department of Veterinary Microbiology and Pathology, Washington State University, Pullman, WA 99164, USA

**Keywords:** whole-proteome sequences, Alphaproteobacteria, bacterial pathogens, bacterial phenotypes, *pClust*

## Abstract

Thousands of whole-genome and whole-proteome sequences have been made available through advances in sequencing technology, and sequences of millions more organisms will become available in the coming years. This wealth of genetic information will provide numerous opportunities to enhance our understanding of these organisms including a greater understanding of relationships among species. Researchers have used 16S rRNA and other gene sequences to study the evolutionary origins of bacteria, but these strategies do not provide insight into the sharing of genes among bacteria via horizontal transfer. In this work we use an open source software program called *pClust* to cluster proteins from the complete proteomes of twelve species of Alphaproteobacteria and generate a dendrogram from the resulting orthologous protein clusters. We compare the results with dendrograms constructed using the 16S rRNA gene and multiple sequence alignment of seven housekeeping genes. Analysis of the whole proteomes of these pathogens grouped *Rickettsia typhi* with three other animal pathogens whereas conventional sequence analysis failed to group these pathogens together. We conclude that whole-proteome analysis can give insight into relationships among species beyond their phylogeny, perhaps reflecting the effects of horizontal gene transfer and potentially providing insight into the functions of shared genes by means of shared phenotypes.

## 1. Introduction

Because 16S rRNA is highly conserved and the rate of nucleotide changes is slow and predictable, it has become the first-line tool for inferring bacterial phylogeny [1]. There are, however, a number of reports cautioning that it is impossible to explain all bacterial evolution using a single gene. As a result, a number of other approaches have been developed that generally confirm the results of the 16S rRNA tool or else introduce refinements to them (e.g., see [2,3,4,5,6,7]). However, there are other relationships that cannot be determined from one or even a handful of genes. For example, we know that genes can be shared among bacteria by means of horizontal gene transfer (HGT), which gives rise to shared phenotypes. Because of the unpredictability of HGT, it is impossible to precisely identify its phylogenic impact, but it is possible to capture a snapshot of its effects at a given time and to glean some useful information regarding the transmission of genes among different species by examining whole-genome or whole-proteome sequences.

The advent of modern sequencing technology has provided us with an unprecedented opportunity to examine relationships among species. Thousands of whole genomes and whole proteomes are now available, and millions should become available in the coming years. Current methods for studying phylogenic relationships at the genome level are mainly based on sequence alignment and analysis of a large number of conserved genes [8,9,10,11,12], comparison of the presence or absence of homologous genes [13,14], or comparisons of whole genomes [15,16,17,18,19,20]. In this work we use a method introduced in [21] to cluster proteins from twelve whole proteomes from the Alphaproteobacteria class within the Proteobacteria phylum. We compare results with the well-established 16S rRNA phylogeny for the twelve species as well as with results obtained using seven housekeeping genes [22]. 

Alphaproteobacteria species were chosen for this study because they are relatively well characterized taxonomically using traditional methods and a number of complete genome sequences are available [23]. Moreover, many genera (e.g., *Rickettsiales*, *Brucella*, and *Bartonella*) are major animal pathogens. Twelve species of Alphaproteobacteria were selected from published work [24], including four animal pathogens, and their whole proteomes downloaded from NCBI (Table 1). Trees were constructed using the 16S rRNA sequences, seven housekeeping genes (see Table 1 of [22]), and the whole-proteome sequences. For the 16S rRNA trees we used both unweighted and Weighbor-weighted bootstrapping with the neighbor joining method. We confirmed the overall 16S rRNA tree structure using Weighbor-weighted bootstrapping with the maximum likelihood and maximum parsimony methods. For the housekeeping genes we used multiple sequence alignment followed by tree construction using minimum evolution, neighbor joining, and UPGMA. For the whole-proteome method we used two different distance metrics, Euclidean and Jaccard, with neighbor joining. The whole-proteome approach uses the open-source software program *pClust* [25] to cluster all orthologous proteins into groups, which, as described in [21], gives significantly better clustering results than clustering via BLAST [26]. 

**Table 1:** Twelve Alphaproteobacteria genomes used in this study.

Organism	Accession Number	Genome Size (bp)	Number of CDS
*Mesorhizobium loti* MAFF303099	NC_002678	7,036,071	6,743
*Sinorhizobium meliloti* 1021	NC_003047	3,654,135	3,359
*Bradyrhizobium japonicum* USDA 110	NC_004463	9,105,828	8,317
*Rhodopseudomonas palustris* CGA009	NC_005296	5,459,213	4,813
*Bartonella quintana* str. Toulouse	NC_005955	1,581,384	1,142
*Bartonella henselae* str. Houston-1	NC_005956	1,931,047	1,488
*Rickettsia typhi* str. Wilmington	NC_006142	1,111,496	837
*Beijerinckia indica* subsp. indica ATCC 9039	NC_010581	4,170,153	3,569
*Brucella melitensis* ATCC 23457, Chrs 1	NC_012441	2,125,701	2,063
*Rhizobium leguminosarum* WSM1325	NC_012850	4,767,043	4,565
*Methylobacterium extorquens* DM4	NC_012988	5,943,768	5,594
*Rhodomicrobium vannielii* ATCC 17100	NC_014664	4,014,469	3,565

## 2. Results and Discussion

**Figure 1:**
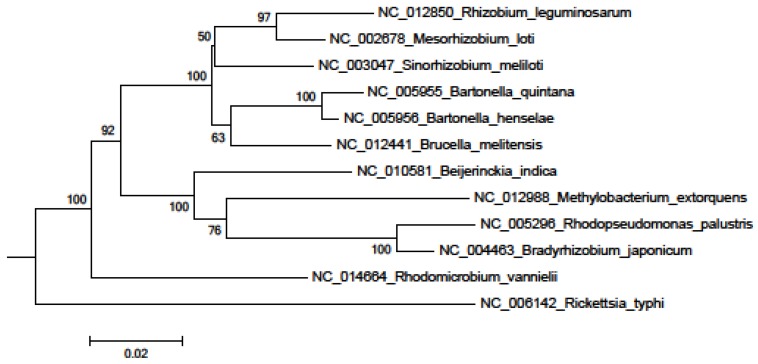
16S rRNA Weighbor-weighted neighbor-joining tree for 12 Alphaproteobacteria.

**Figure 2:**
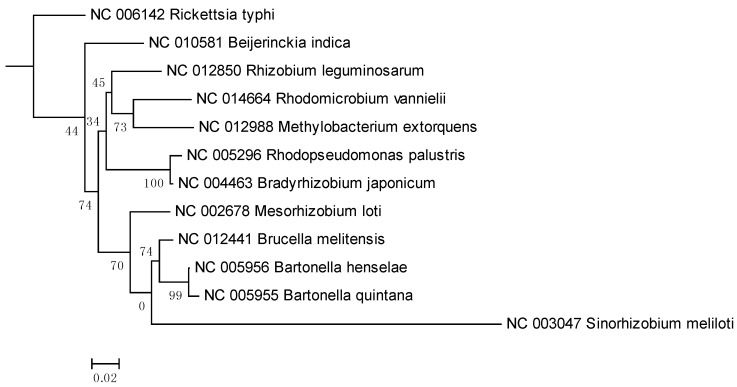
16S rRNA Weighbor-weighted maximum likelihood tree for 12 Alphaproteobacteria.

**Figure 3:**
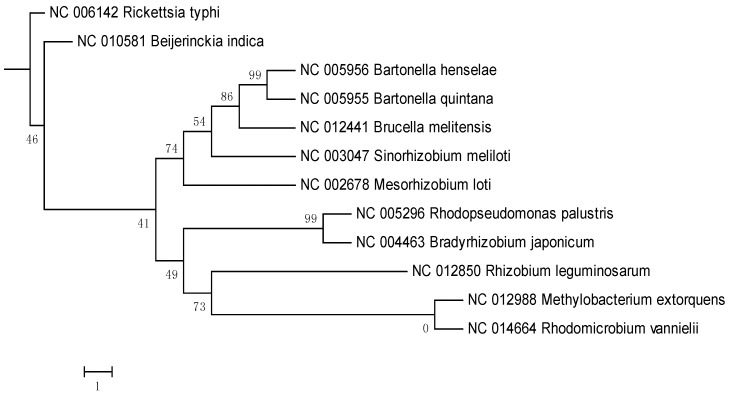
16S rRNA Weighbor-weighted maximum parsimony tree for 12 Alphaproteobacteria.

**Figure 4:**
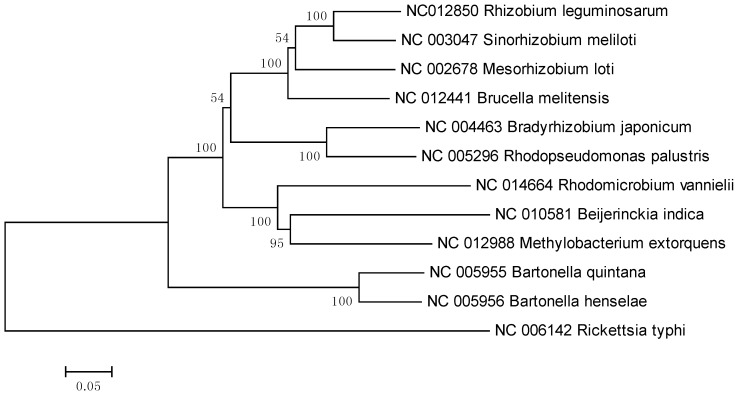
Neighbor-joining tree obtained using multiple sequence alignment of seven housekeeping genes for 12 Alphaproteobacteria.

**Figure 5:**
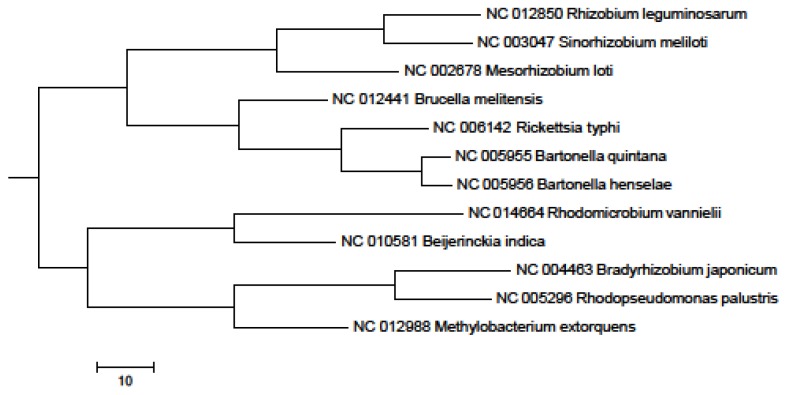
Euclidean distance tree for 12 Alphaproteobacteria using whole-proteome sequences.

**Figure 6:**
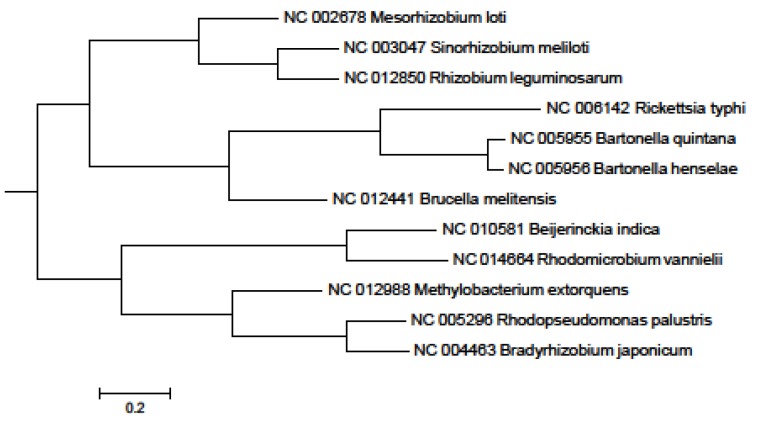
Jaccard distance tree for 12 Alphaproteobacteria using whole-proteome sequences.

In the 16S rRNA results, the lower parts of the unweighted (results not shown) and Weighbor-weighted (Figure 1) neighbor-joining bootstrapped trees are very similar, but there is a slight difference in the upper part for *Sinorhizobium meliloti*. We used the maximum likelihood and maximum parsimony methods (Figure 2 and Figure 3, respectively) for confirmation. While there are differences among the results, these differences are unimportant for our comparison, and the overall structure agrees with our expectations: The eleven species from the order Rhizobiales are clustered together, and the twelfth species from the order Rickettsiales forms a singlet cluster. Moreover, these results are consistent with those obtained using more sophisticated techniques (see, for example, [27,28]). Figure 4 shows the neighbor-joining tree results using multiple sequence alignment of seven housekeeping genes. As with the 16S rRNA results, the Rickettsiales species forms a singlet cluster. The minimum evolution and UPGMA trees (results not shown) gave similar results. The whole-proteome results for Euclidean and Jaccard distance metrics have very similar topologies (Figure 5 and Figure 6, respectively). They recapitulate the Rhizobiales topology, clustering the soil-borne species of the families Brucellaceae, Rhizobiaceae, and Phyllobacteriaceae separately from the other soil-borne Rhizobiales. There is, however, a striking difference between both the 16S rRNA and housekeeping gene results and the whole-proteome results; the Rickettsiales species, *Rickettsia typhi*, is clustered together with *Bartonella quintana*, *Bartonella henselae*, and also with *Brucella melitensis* rather than forming an outlying singlet cluster as it does with the 16S rRNA and housekeeping gene trees. These four species are pathogens causing, respectively, murine typhus, trench fever, cat scratch disease, and Brucellosis, whereas the other eight species are not pathogens.

It has been challenging to determine the interrelationships among the different Alphaproteobacteria families on the basis of the 16S rRNA gene [29,30]. The results described above indicate that the use of whole-proteome sequences has the potential to illuminate fine-scale interrelationships—e.g., the clustering of patent pathogens that are otherwise segregated when limited sequence data sets are compared. This cluster of pathogens may reflect the impact of horizontal gene transfer in conferring phenotypic traits to otherwise unrelated species. As such, it may provide insight into the function of shared genes. For example, one interesting avenue of study would be to identify the protein clusters in which the four pathogens have proteins in common but most of the non-pathogens do not and perform experimental lab work to determine whether any of these genes contribute to pathogenicity. The possibility of shared genes that contribute to pathogenicity is intriguing given that the genome—and, hence, proteome—of *R. typhi* has been reduced over time as a result of interactions between host and pathogen [31]; this is also true of the two species of *Bartonella* with which *R. typhi* is most closely clustered.

## 3. Experimental

Twelve species of Alphaproteobacteria were selected from published work [24], eleven from one order and the twelfth from another order; these included four pathogens. 16S rRNA gene sequences were downloaded from [32]. The complete genome sequences for these twelve species were downloaded from [33] (Table 1). As there are many strains for each species—e.g., there were five different strains of *Brucella melitensis*—we randomly selected one to serve as the species representative.

Two different methods were applied to build the 16S rRNA tree for the twelve species. One was the unweighted, neighbor-joining bootstrapped consensus tree (including bootstrap values) and the other was the Weighbor-weighted neighbor-joining tree constructed using the tree builder tool of the Ribosomal Database Project (RDP) [34]. For the unweighted method, the neighbor-joining tree was obtained using MEGA5 with 500 bootstrapping iterations based on the results of multiple sequence alignment from ClustalW with default settings [35]. The Weighbor-weighted consensus tree was implemented in the manner described in [24]. Weighbor is a weighted version of neighbor joining that assigns much less weight to longer distances in the distance matrix. The weights are based on variances and covariances expected in a simple Jukes-Cantor model [36].

Seven classic housekeeping genes were downloaded from NCBI from a *Brucella abortus* NC_006932 proteome (*gap*, *aroA*, *glk*, *dnaK*, *gyrB*, *trpE*, and *cobQ*) [22]. BLASTp was used to identify orthologs from each of the twelve Alphaproteobacteria proteomes using an E-value cut-off of <0.001. ClustalW was used to perform multiple sequence alignment of the seven gene sequences for all twelve species, and the results were used with MEGA5 to construct minimum evolution, neighbor-joining, and UPGMA trees with bootstrapping using 100 iterations.

More than 46,000 proteins were extracted from the twelve genomes, and these proteins were clustered into orthologous groups using *pClust* [25]. The details of this approach are given in [21], but briefly, *pClust* uses the Smith-Waterman algorithm, which guarantees the optimal solution, to perform pairwise comparison on a subset of the total number of protein sequences used as input—in our case the >46,000 genome proteins—obtained after filtering has occurred. Importantly, *pClust* is much more sensitive than BLAST. In fact, in an unpublished study, BLAST missed 14% of the clustered pairs obtained using *pClust*. The filtering step removes sequences that are shorter than the window size (one of the configuration parameters) and sequence pairs that do not share at least one exact match of length greater than or equal to the cut-off (another of the configuration parameters that contributes most of the filtering), and the strength of filtering is determined by the two parameter settings in the configuration file. The default settings were used except for ExactMatchLen, which was set to 4 rather than the default value of 7. The smaller value provides less stringent filtering so that more proteins are compared. A total of 6,325 orthologous protein groups (defined as having at least two proteins) were identified by *pClust*. A binary matrix 12 × 6,325 in size, each row representing one of the twelve species, was formed with a 1 or 0 indicating presence or absence, respectively, of a given genome protein in each of the 6,325 groups. This binary matrix was used to construct the tree using two different distance metrics, the Jaccard distance metric, which is used for binary matrices, and the Euclidean distance metric, which is a standard distance metric, and neighbor joining was used to obtain the final trees.

## 4. Conclusions

While it is intuitive that whole-genome and whole-proteome sequences should help to clarify relationships among organisms, until recently no satisfying approach has been proposed to efficiently use these data. In this work, we examined the relationships among twelve Alphaproteobactera species beyond that of their phylogeny. We constructed trees using 16S rRNA genes, seven housekeeping genes, and a whole-proteome approach, which clusters proteins from all the proteomes. Comparison of the trees shows that the whole-proteome approach reflects phenotypic traits, with all pathogens clustered in one group as opposed to the 16S-rRNA and housekeeping-genes trees in which the Rickettsiales species appears as a singlet cluster. We assume that the clustering of the pathogens represents the effects of shared genes in creating phenotypic relationships.
